# Health Impact Assessment of Air Pollution in São Paulo, Brazil

**DOI:** 10.3390/ijerph13070694

**Published:** 2016-07-11

**Authors:** Karina Camasmie Abe, Simone Georges El Khouri Miraglia

**Affiliations:** Instituto de Ciências Ambientais, Químicas e Farmacêuticas (ICAQF), Laboratório de Economia, Saúde e Poluição Ambiental, Universidade Federal de São Paulo–UNIFESP, Rua São Nicolau 210, Diadema, São Paulo CEP 09913-030, Brazil; kaabe36@yahoo.com.br

**Keywords:** air pollution, Health Impact Assessment, public health, ozone, particulate matter, air quality, health management, Brazil, economic costs

## Abstract

Epidemiological research suggests that air pollution may cause chronic diseases, as well as exacerbation of related pathologies such as cardiovascular and respiratory morbidity and mortality. This study evaluates air pollution scenarios considering a Health Impact Assessment approach in São Paulo, Brazil. We have analyzed abatement scenarios of Particulate Matter (PM) with an aerodynamic diameter <10 μm (PM_10_), <2.5 μm (PM_2.5_) and ozone concentrations and the health effects on respiratory and cardiovascular morbidity and mortality in the period from 2009 to 2011 through the APHEKOM tool, as well as the associated health costs. Considering World Health Organization (WHO) standards of PM_2.5_ (10 μg/m^3^), São Paulo would avoid more than 5012 premature deaths (equivalent to 266,486 life years’ gain) and save US$15.1 billion annually. If São Paulo could even diminish the mean of PM_2.5_ by 5 μg/m^3^, nearly 1724 deaths would be avoided, resulting in a gain of US$ 4.96 billion annually. Reduced levels of PM_10_, PM_2.5_ and ozone could save lives and an impressive amount of money in a country where economic resources are scarce. Moreover, the reduced levels of air pollution would also lower the demand for hospital care, since hospitalizations would diminish. In this sense, Brazil should urgently adopt WHO air pollution standards in order to improve the quality of life of its population.

## 1. Introduction

Several epidemiological studies have demonstrated a strong association between acute and chronic exposures to Particulate Matter (PM) with an aerodynamic diameter <10 μm (PM_10_) or <2.5 μm (PM_2.5_) and cardiovascular diseases [1,2,3,4,5]. In this context, the World Health Organization (WHO) and many national governments have set health-based Air Quality Standards (AQS) for PM and ozone due to the high evidence that these pollutants could lead to several outcomes that impact health. The technical analyses of the benefits of air quality programs or public policies regarding pollutants concentration have become an increasingly important component of national decision-making.

PM is a mixture of solid and liquid particles and its composition and size may vary; in urban areas, its concentration is a matter of concern. PM is derived from emissions from mobile sources such as cars, motorbikes, buses and trucks, and also from stationary sources such as heating furnaces, power plants and factories [6]. Ozone is one of the most toxic components of the photochemical air pollution mixture and it has been associated with increases in mortality and hospital admissions due to respiratory and cardiovascular disease [7].

Although air pollution is an intrinsically urban issue, numerous international studies have shown evidence that lowering air pollution exposure leads to less adverse health effects [8,9,10]. In an era in which the importance of sustainable development and its impact on environment and public health has gained even more recognition worldwide, this outcome has forced policymakers to tackle the problem of air pollution [11]. The authors of a Belgian study projected that reduced healthcare costs for air pollution levels above the WHO guideline for PM_2.5_ would be €51 million for ischemic heart diseases and heart rhythm disturbances combined [11]. In Iran, Brajer et al. [12], have estimated the annual economic benefits of this reduced risk would be US$378.5 million, if health-based WHO-recommended annual average PM_2.5_ standards were met.

In Brazil, Miraglia and Gouveia [13] have estimated the cost of premature deaths due to air pollution in 29 Brazilian capital cities and the result was a loss of US$1.7 billion annually [13]. Nevertheless, it is necessary to conduct comprehensive studies and pursue diverse research approaches. One of the recommended methodologies that focuses on the issue is the “Health Impact Assessment” (HIA) methodology. According to WHO, HIA is “a combination of procedures, methods, and tools used to evaluate the potential health effects of a policy, program or project. Using qualitative, quantitative and participatory techniques, HIA aims to produce recommendations that will help decision makers and other stakeholders make choices about alternatives and improvements to prevent disease/injury and to actively promote health” [14].

HIA methodology is derived from the WHO-HIA general method [15] and quantifies the impact of air pollution exposure on health using some successive steps that include selecting concentration-response function, usually expressed as relative risks from epidemiological studies; includes the distribution of effects on the target population; promotes the creation of health indicators and calculates the number of attributable health cases as mortality and morbidity cases [16]. This recent methodology has already been used by others studies combined with one or two predictive scenarios of air pollution [17,18,19]. In Brazil, there are no studies involving HIA and associated costs with reducing pollution levels scenarios. In this study, we aimed to determine potentially avoided negative health effects and costs resulting from an abatement of air pollution in São Paulo, Brazil.

## 2. Materials and Methods

### 2.1. Study Period and Areas

The HIA was performed in São Paulo using the APHEKOM model. This model has already been used in several studies to improve decision-making on air pollution [17,19,20,21]. A common study period, 2009–2011, was chosen based on data availability. A study area was defined according to pollutant database collections in order to ensure that average pollutant levels measured at fixed monitors could be considered acceptable statistical representations of the population’s average exposure.

### 2.2. Population and Health Data

Data on the population of São Paulo and its health were collected from public authorities (DATASUS and IBGE) [22,23]. Mortality data were selected by the main cause of death of the residents living in the study area. Hospitalization data refer to public hospitals only.

### 2.3. Exposure Assessment

In São Paulo study area, data were provided by the local air quality monitoring network by CETESB [24], who has 11 monitoring stations. However, not all the stations provide measurements of all the pollutants. We have chosen stations that had measured PM_10_ and ozone concentrations and we have analyzed the trend and variability of pollutant´s concentration. Because they were similar for all stations (e.g., when pollution levels increase in the central area, there is a proportional increase in the suburbs), the values obtained at stations with available records could be averaged and considered indicative of the citywide status.

The ozone levels were measured using the standard reference ultraviolet absorption method [24] Ozone exposure indicator was the daily maximum 8 h mean (daily maximum of the 8 h running means) of all selected stations, including only those years with less than 25% missing data. Regarding PM measurements, the network stations used the beta radiation method. For São Paulo, PM_2.5_ measures were not available. In this sense, our group estimated the PM_2.5_ concentration from daily PM_10_ values using a 0.7 conversion factor, as defined in the Apheis project [17,25].

### 2.4. Calculation of the Health Impacts

In this study, we evaluated the health benefits that could be achieved if pollutant concentrations were decreased. Regarding PM_10_ and ozone, the short-term exposure was estimated and the health impact was computed as follows [17] (Equation (1)):

Δy = y_0_ (1 − e^−βΔx^) (1)

where:

Δy—means the decrease in the annual number of hospitalizations or deaths associated with the reduction of pollutant concentrations.
y_0_—means the baseline health outcome, in annual number of deaths or hospitalizations.
β—is the coefficient of the concentration response function.
Δx—is the decrease in the pollutant concentration in a specific scenario, shown in μg/m^3^.

The two scenarios were built as follows: (1) a decrease in the annual mean by a fixed amount of 5 μg/m^3^ and (2) a decrease of the annual mean down to the annual WHO-Air Quality Standards (WHO-AQS). The WHO-AQS values of pollutants’ concentration were 20 μg/m^3^ for PM_10_ and 10 μg/m^3^ for PM_2.5_. The WHO-AQS (100 μg/m^3^) for daily ozone concentration was applied in order to assess the short-term impacts of ozone. Regarding PM_2.5_ long-term health effects exposure, we applied a standard abridged life table methodology as described by Pascal et al. [17].

The results of the predictive scenarios were represented as the number of avoided deaths in each scenario and the additional life expectancy at age 30. In this study, São Paulo’s population age 30 and older is 6,004,495.

The detailed methodology, concentration response functions and equations were available at “guidelines for performing an HIA of the health impacts of urban air pollution” [26], and all databases were populated with São Paulo data using Microsoft Excel^®^ spreadsheets developed by the APHEKOM software, already in use in studies elsewhere [17].

### 2.5. Economic Valuation

#### 2.5.1. Mortality

Recently, there has been a growing interest in the creation of methodologies to express trade-offs between mortality and economic costs [17]. A way to achieve the value of postponed deaths is termed Value of a Statistical Life (VSL). An important key finding is that the VSL depends on health outcomes and the characteristics of the risk of death, considering relevant factors such as age, time between exposure and death and nature of the underlying risk [27,28].

Therefore, to achieve a standard indicator, we applied the Disability-Adjusted Life Years (DALY) method to estimate the burden of disease due to air pollution in São Paulo [29]. The DALY method was elaborated by the World Bank and WHO as an initiative to standardize a worldwide health cost estimative using a single measure of health outcome [30]. This method involves two components: the first component refers to Years of Life Lost due to premature death (YLLs) and the second component refers to Years of Life Lived with Disability (YLDs) [30]. In this work we only assess the YLL component of DALY. The total amount of YLL can be expressed in economic terms by utilizing the concept of Value of Life Year (VOLY), which was considered to be €50,000 [31]. The conversion factor for American dollars is 1.13280812 (22 June 2016) which means an equivalent VOLY of US$56,640.41.

#### 2.5.2. Morbidity

In relation to morbidity costs, analyses considered hospitalization expenditures, so we used the approach of the standard cost of illness. It consists of applying a unique economic value that combines the direct and indirect costs of each hospitalization [17]. However, some intangible costs cannot be assessed: for example, pain and suffering. The cardiac and respiratory hospitalizations’ direct costs were considered from an average cost per day and an average length of hospital admission. These unit hospitalization costs and average number of hospitalization days in the city of São Paulo were also obtained from DATASUS.

Thus, the morbidity evaluation was estimated as follows (Equation (2)):

Ch = Vi × Nd × Nc(2)

where:

Ch = Cost of hospitalization
Vi = unit value of a daily admission
Nd = average number of admission days due to a certain disease
Nc = number of cases due to a certain disease

The present study considered the average number of admission days due to respiratory diseases in São Paulo to be 8.1 and those due to cardiac diseases to be 8.8 as provided by DATASUS. The unit value of a daily admission was US$ 366.49 for a respiratory admission and US$ 837.72 for a cardiac admission in São Paulo (according to DATASUS). Finally, the number of cases due to a certain disease attributable to ozone and PM_10_ can be found in Table 3 and Table 4 in the Results section.

## 3. Results

### 3.1. Characteristics of the Local of Study and Population

São Paulo is located in Southeastern Brazil, in southeastern São Paulo State. The city is located at an average elevation of around 799 m (2621 ft.) above sea level. According to the 2010 census of Brazilian Statistic and Geography Institute (IBGE), there were 11,245,983 people residing in São Paulo, of which 7,996,444 are between 16 and 64 years old and 914,570 are older than 65. The annual number of hospitalizations for all ages due to cardiac causes varied from 58,148 in 2009 to 63,250 in 2011. The respiratory hospitalizations for all ages varied from 57,714 in 2009 to 57,599 in 2011 (Table 1).

### 3.2. Descriptive Analysis of Pollutants

The maximum daily 8-h ozone value was 256 µg/m^3^ and the minimum was 10 µg/m^3^ for the period 2009–2011. The daily average ± standard deviation (SD) was 83 µg/m^3^ ± 36 µg/m^3^ with median 78 µg/m^3^. For the PM_10,_ the maximum daily value was 131 µg/m^3^ and the minimum was 8 µg/m^3^ for the period 2009–2011. The daily average was 36 µg/m^3^ ± 17 µg/m^3^ (SD), with median 32 µg/m^3^. The values are summarized in Table 2.

### 3.3. Short-Term Impacts of PM_10_ Exposure on Morbidity

Considering the period between 2009 and 2011, if a PM_10_ WHO level of 20 μg/m^3^ had been reached, São Paulo would have avoided more than 1500 cardiovascular and respiratory hospitalizations annually. A decrease by 5 μg/m^3^ would have avoided more than 500 hospitalizations (Table 3) caused by respiratory and cardiovascular diseases.

### 3.4. Short Term Effects of Ozone Exposure on Mortality and Morbitidy

Regarding ozone levels, compliance with the WHO standard of 100 μg/m^3^ for the maximum daily 8 h ozone mean would have resulted in the avoidance of more than 50 respiratory hospitalizations annually in the population aged 15 years or older (Table 4) and could have postponed 152 deaths (non-external mortality) over the short term. In addition, if the ozone mean concentration had been reduced by 5 μg/m^3^, it would have resulted in an annual decrease of more than 34 respiratory hospitalizations and more than 98 deaths from non-external mortality (Table 4).

### 3.5. Long-Term Impacts of PM_2.5_ Chronic Exposure on Mortality

In São Paulo, if the PM_2.5_ WHO standards had been reached (10 µg/m^3^), the annual number of postponed deaths would have been more than 5000 annually and life expectancy would have increased by 15.8 months (Table 5). That is equivalent to a 266,486 life years’ gain and US$ 15.1 billion annually. If São Paulo could even diminish the mean of PM_2.5_ by 5 μg/m^3^, nearly 1725 deaths would be postponed annually and the population would gain more than 5 months in life expectancy, resulting in a gain of US$ 4.96 billion.

## 4. Discussion

São Paulo is the largest city in Brazil and the capital of the state of São Paulo. It has the world's seventh largest Gross National Product and population. It exerts strong regional influence in commerce, finance, culture and entertainment and has an impressive international influence [32]. As the largest city in Brazil, São Paulo has high levels of air pollution and consequently several associated health outcomes.

Considering the analyzed period (2009 through 2011), the ambient levels of PM_10_, PM_2.5_ and ozone were higher than those recommended by WHO to protect public health. The chronic effects of air pollution have been considered by estimating the number of years of life lost due to long-term exposure to air pollution. In this sense, morbidity was considered only for acute effects of air pollution (e.g., cardiorespiratory hospitalizations, asthma or bronchitis symptoms) [19,33]. However, nowadays it is known that chronic morbidity due to air pollution also has impacts on health and the healthcare system in addition to acute effects [19].

Our findings and predictive scenarios showed the potential economic gain if a marked and sustained reduction in ambient ozone and PM levels could be reduced to WHO compliance levels. The more important health burden was considered to be the impacts of chronic exposure to PM_2.5_ [4,34]. Regarding this pollutant, levels complying with the WHO guideline of 10 μg/m^3^ in annual mean would add up to 15.8 months of life expectancy, corresponding to a postponement of 5012 deaths and a gain of US$15.1 billion annually, excluding savings on health expenditures, absenteeism and intangible costs such as quality of life and life expectancy. Mortality remains our best choice for health outcomes, as it is robust, easy to obtain from existing high-quality records in São Paulo and not subject to misclassification [17]. Nevertheless, we also used hospitalization data; but they are less robust and more heterogeneous.

The decrease in quality of life and life expectancy due to air pollution is a common phenomenon. Recently, Perez et al. [19] have observed the causal relationship between asthma and near road traffic-related pollution exposure in 15% of all episodes of asthma symptoms. In the same study, they found similar patterns for coronary heart diseases in older adults. Moreover, in the case of cardiovascular mortality and myocardial infarction, there is also a large amount of evidence that acute and chronic health outcomes are related [5,35,36].

In a more comprehensive review of the new evidence linking PM exposure with cardiovascular disease, the American Heart Association focused on the clinical implications for researchers and healthcare providers. Studies have shown that exposure to PM_2.5_ over a few hours to weeks can trigger cardiovascular disease-related mortality and nonfatal events; and longer-term exposure could increase the adverse effects on microvascular functions and the risk for cardiovascular mortality [35,37]. However, reductions in PM levels are associated with decreases in cardiovascular mortality within a period as short as a few years [35]. In this sense, the gain in quality of life and health outcomes due to the reduction of air pollution levels could be perceived in a few years by the population, becoming essentially the implementation of more stringent public policies on air quality in Sao Paulo and other large Brazilian cities.

Pascal et al. [17] conducted a large APHEKOM study in 25 European cities and the main economic outcome regarding mortality benefits of complying with the WHO standards of 10 μg/m^3^ for PM_2.5_, would represent an average gain of €31 billion euros. This value is underestimated due to the limitations of the Pascal study (which are the same limitations of the present study, since both studies have considered the analysis of only three pollutants and use of the average concentration exposure for the population). Further, our study underestimated the impact and costs of chronic exposure to PM and ozone, since the variation of exposure within the city is very frequent, notably in relation to road traffic.

PM adverse health effects could be aggravated by exposure to another pollutant, which is a component of the photochemical air pollution mixture: ozone. Some epidemiologic research works have suggested significant outcomes to ozone exposure on human health [38,39,40]. Some health effects of ozone can include lung epithelial damage and inflammatory response [41,42], including decreases in mucociliary clearance [38]. All these effects can cause susceptibility to infections. In a European study, researchers have obtained an increase in risk between 0.33% and 1.13% on the total daily number of deaths, cardiovascular deaths and respiratory deaths, respectively, due to an increase of 10 µg/m^3^ in the 1 h or 8 h ozone concentration exposure [40]. In the present study, predictive scenarios regarding ozone concentration decrease can prevent more than 50 annual respiratory hospitalizations in the 15-years-or-older population. This number is underestimated, since we can have a higher level of a non-hospitalization care due to respiratory symptoms; and in this study we did not estimate the hospitalization of patients under 15 years old. Considering non-external mortality, even an ozone level decrease of 5 μg/m^3^ could avoid almost 100 premature deaths and compliance with WHO standards could postpone more than 150 deaths annually in the adult population. All these results suggest the necessity of better public policies regarding air quality in large cities in Brazil.

Recently, a Brazilian study aimed to evaluate the economic impact of health events associated with current levels of air pollution in 8 cities that have air monitoring and 29 more cities without monitoring stations. The study concluded that Brazilian metropolitan regions have annually high economic losses, reaching almost US$ 3 billion, due to air pollution mortality [13]. The present study shows the potential health and economic benefits for São Paulo through compliance with WHO guidelines, evidencing the amount of avoided adverse health effects (about 5000 deaths) and associated monetary gains (around US$ 15 billion). It is understood that this economic valuation represents an efficient basis for the formulation of air pollution mitigation policies, since valuing health effects is a way to create indicators to subsidize better decision-making by stakeholders. In the present study, we presented an overview not only about past mortality, but also about future scenarios of interventions aiming to reduce pollutant concentrations, such as investing in cleaner fuels and/or expanding the infrastructure of the subway system, among other options. In an era in which the importance of sustainable development and its impact on the environment and public health has gained more international recognition, this outcome has forced policymakers to tackle the problem of air pollution [11].

As the main source of air pollution in major city centers is transportation, there is an urgent need to regulate pollutants from major sources such as transportation, industry and energy production in accordance with the most stringent standards. In this sense, the Brazilian government should aim to use all available resources to decrease air pollutant concentration in urban centers, as this means saving lives and increasing the quality of life of thousands of people; likewise, it means millions of dollars in gains to the Brazilian economy.

Curbing the negative health effects of air pollution pays dividends. This study shows a suggestive approach that gives decision makers across several Brazilian ministries a compelling reason to act. Avoiding adverse health effects saves money, and, more importantly, lives.

In this sense, Brazil should urgently adopt WHO air pollution standards in order to improve the quality of life of its population.

## 5. Conclusions

This study is a pioneering one in Brazil concerning the HIA approach for evaluating scenarios of air pollution reduction. It has demonstrated the health and monetary benefits that could be obtained from implementing effective policies for air pollution control in São Paulo and ensuring compliance with them over time. This is the first time using an APHEKOM modelling application outside Europe and it will be very useful for improving public health policies concerning air pollution threats in developing urban centers. As a limitation of this work, we can consider the use of international concentration-response functions instead of a national one, due to a lack of national cohort and time series studies. Another aspect is the use of a European VOLY. First, in Brazil there is no available data regarding national VOLY and second, our goal is to make this study comparable to other European studies involving APHEKOM and also support national public policies, which are heavily dependent on monetary values.

Reduced levels of PM_10_, PM_2.5_ and ozone could save lives and an impressive amount of money in a country where economic resources are scarce. Moreover, reduced levels of air pollution would also prevent the demand for hospital space, since there would be diminished hospitalizations.

## Figures and Tables

**Table 1:** The annual mean number of hospitalizations due to cardiac and respiratory causes and cardiac mortality for the period 2009–2011 in São Paulo, Brazil.

Health Outcome	ICD *-10 Codes	Age Group	Annual Mean Number	Annual Rate per 100,000
Total Non-external Causes Mortality	A00-R99	All Ages	63,990	569
Cardiac Hospital Admissions	I00-I52	All Ages	61,548	547
Respiratory Hospital Admissions	J00-J99	All Ages	57,824	514
Total mortality	A00-Y98	>30	60,069	1000

* ICD: International Classification of Diseases.

**Table 2:** Descriptive statistics of pollutant concentration.

Pollutant	Daily Mean	Standard Deviation	Minimum	Maximum
	(μg/m^3^)	(μg/m^3^)	(μg/m^3^)	(μg/m^3^)
**PM_2.5_ (daily average)**	21	10	5	78
**Ozone (daily 8 h max)**	83	36	10	256
**PM_10_ (daily average)**	36	17	8	131

Particulate Matter (PM) with an aerodynamic diameter <10 μm (PM_10_) or <2.5 μm (PM_2.5_).

**Table 3:** Potential health benefits of reducing daily PM_10_ levels on hospitalizations.

Scenarios	Respiratory Hospitalizations		Cardiac Hospitalizations		Monetary Valuation (US$ Millions)
	Annual Number of Avoided Cases	Annual Number of Avoided Cases Per 100,000	Annual Number of Avoided Cases	Annual Number of Avoided Cases Per 100,000	
Decrease by 5 μg/m^3^	326.8	2.91	183.8	1.63	2.32
Decrease to 20 μg/m^3^	1016.5	9.04	573.4	5.10	7.25

**Table 4:** Potential health benefits of reducing daily ozone levels in terms of hospitalizations over the short term and total non-external mortality.

Scenarios	Respiratory Hospitalizations (15–64)		Respiratory Hospitalizations (>64)		Total Non-External Mortality		Monetary Valuation (US$ Millions)
	Annual Number of Avoided Cases	Annual Number of Avoided Cases Per 100,000	Annual Number of Avoided Cases	Annual Number of Avoided Cases Per 100,000	Annual Number of Avoided Deaths	Annual Number of Avoided Deaths Per 100,000	
8 h-max daily values >100 μg/m^3^ = 100 μg/m^3^	11.85	0.15	41.68	4.56	152.38	1.36	24.9
Decreased by 5 μg/m^3^	7.69	0.10	27.08	2.96	98.95	0.88	17.1

**Table 5:** Potential health and economic benefits of reducing annual PM_2.5_ levels over the long term in terms of total non-external mortality.

Scenarios	Annual Number of Deaths Avoided	Annual Number of Deaths Avoided Per 100,000	Gain in Life Expectancy (months)	Life Years Gain	Monetary Valuation (US$) *
**Decrease by 5 μg/m^3^**	1724.8	28.7	5.2	87,548.9	4,958,805,241
**Decrease to 10 μg/m^3^**	5012.2	83.5	15.8	266,486.3	15,093,892,225

* Monetary valuation is based on Life Years Gain.

## Abbreviations

The following abbreviations are used in this manuscript:

DALY	Disability-Adjusted Life Years
HIAICD	Health Impact AssessmentInternational Classification of Diseases
PM	Particulate matter
PM_10_	Particulate matter with diameter <10 µm
PM_2.5_	Particulate matter with diameter <2.5 µm
SD	Standard Deviation
VOLY	Value of Life Years
VSL	Value of a Statistical Life
WHO	World Health Organization
WHO-AQS	World Health Organization-Air Quality Standards
YLD	Years Lived with Disability
YLL	Years of Life Lost
